# Epidemiology of Gastric Cancer in the Gangetic Areas of West Bengal

**DOI:** 10.1155/2013/823483

**Published:** 2013-10-23

**Authors:** Ashis Kumar Saha, Somnath Maitra, Subhas Chandra Hazra

**Affiliations:** Department of General Medicine, K.P.C. Medical College, 1F Raja S.C. Mullick Road, Jadavpur, Kolkata, West Bengal 700 032, India

## Abstract

There is marked geographical variation in the distribution and incidence of stomach cancer. We tried here to describe the pattern of relationships of age, sex, religion distribution, symptom profile, histological subtypes and *Helicobacter pylori* (*H. pylori*) infection with gastric cancer in Gangetic West Bengal. This study was done over a period of five years (2006–2010). The patients residing in the Gangetic areas of West Bengal presenting with upper gastrointestinal symptoms underwent UGI endoscopy. Among gastric cancer patients, demographic characteristics, symptomatology, macroscopic and histologic lesions and *H. pylori* status were analyzed. At confidence level 95%, “*Z*” and “*P*” value were calculated to find significance. Among 23851 patients underwent UGI endoscopy, 14106 were males, 9745 females, 17889 Hindus and 5962 Muslims. Among 462 gastric cancer patients, Male : Female 2.7 : 1, Hindus : Muslim 3 : 1, abdominal pain, indigestion, and weight-loss were commonest presentations. Antrum was the commonest site whereas ulceroproliferative type was commonest type. *H. pylori *positivity was 80.89% in adenocarcinoma with statistically significant relation with intestinal type. In future, our target will be to modify risk factors; it will need further demographic studies and analysis, so that we can detect it earliest.

## 1. Introduction

The incidence of gastric carcinoma has fallen dramatically in the last 50 years, but according to IARC-Globocan 2008, it is the third commonest cause of cancer death after lung and liver cancer in male and after breast and lung cancer in female in the world. It is still the 2nd and 4th most common cancer in males and females, respectively [1, 2]. Interestingly there is a marked geographical variation in stomach cancer. Highest incidence rates were reported in Japan, China, Eastern Europe and a few areas of Latin America and low rise in North America, India, Philippines, most countries in Africa, some Western European countries, and Australia previously. Globally, smoking, tobacco chewing, and alcohol are the risk factors for gastric cancer [3]. In India, there are strong associations between them. Histologically, there are three subtypes of gastric adenocarcinoma-intestinal, diffuse, and indeterminate or mixed type. Of those, intestinal subtype influences the changes in the epidemiological incidence [4]. Case fatality ratio of gastric cancer is higher than other common malignancies, like, colon, breast, and prostate cancer [5]. Worldwide, the well known epidemiological observation in gastric cancer includes the following. (a) If migrants from high risk areas move to low risk areas (China to North America); the incidence rate shows remarkable reduction reaching to almost equal rates as in low risk countries [6]. (b) Preventable by lifestyle modification such as reduced salt intake and increased vegetable and fruits consumption, together with avoidance of smoking and countermeasures against *H. pylori* infection, reduce the risk of gastric cancer [7]. This cancer is usually diagnosed late when the disease has already invaded the lamina propria and musculature, because in early stages, the patient usually complains of trivial and nonspecific symptoms. Hence a new look to epidemiological studies in gastric cancer is necessary to establish new strategies for its early treatment and prevention of recurrence. In this study, we tried to explore the epidemiological characteristics of gastric cancer in Gangetic West Bengal, India, by searching relationships with age, sex, religion distribution, symptom profile, histological subtypes, and association with *H. pylori* infection.

## 2. Materials and Method

We started performing our original and honest study only after getting permission from the ethical committee. This work has been approved by the appropriate ethical committee of our Institution. This study was performed over a period of five years. The study population was from different districts on both sides of Ganges in West Bengal, India (Malda, Nadia, Howrah, Hoogly, North and South twenty-four parganas, and Kolkata). The patients were first screened by the doctors in rural urban health centers, public and district hospitals, nursing homes, and chambers, and then they were sent to hospitals or diagnostic centers of respective districts having facility for upper gastrointestinal endoscopy for evaluation of symptoms related to upper gastrointestinal tract. After getting informed consent from the subjects, demographic data was collected in a proforma containing structured questionnaire from patients and/or patients' party. Then esophagogastroduodenoscopy was performed using 15% xylocaine spray as local anesthesia.

After detecting the lesion, at least eight biopsies were taken from the suspected areas in each patient. Biopsy materials were fixed in ten percent formalin at room temperature and later on processed for histopathology.

Multiple biopsies were also taken from normal looking mucosa adjacent to the growth for determining *Helicobacter pylori* status. For *Helicobacter pylori* testing, rapid urease testing was performed by using HP kit-TM manufactured by Allied Marketing Corporation, Kolkata, with sensitivity of 90% and specificity of 90%. Colour change from yellow to red within the time period of 15–20 minutes confirmed *H. pylori*.

After getting histologically confirmed gastric cancer patients, their demographic characteristics (age, sex, and religion), symptomatology, types of lesions (macroscopic and histologic), and *Helicobacter pylori* status were analyzed.

Statistical methods used as follows.

(1) For significance of percentages, *Z* values (normal deviates) have been calculated. *P* value indicates the maximum probability for a given level of significance.
(2) 95% CI for difference of percentage:
  (1) (p1−p2)±1.96SE(p1−p2), where SE(p1−p2) =  [{  p1(1−p1)n1}+{p2(  1−  p2)n2}].
(3) Chi-square test has been used with two degrees of freedom for Table 5 to show significance of association of affected cases according to types of lesions.

## 3. Results

A total of 23851 patients underwent upper GI endoscopy of which 14106 were males and 9745 were females; 17889 were Hindus and 5962 were Muslims. A total of 462 cases were detected to have gastric malignancy. Demographic data for age, sex, and religion were tabulated in (Tables 1 and 2). Amongst the affected cases (*n* = 462), 340 were males and 122 were females (ratio 2.7 : 1). Religion distribution showed 310 Hindus and 152 Muslims (ratio 3 : 1).  Table 1 also revealed that in more than 40 years, males were predominantly involved in comparison to females.  Table 2 showed that incidence of Muslims was more common in 1st age group. Comparison of presenting symptoms was shown in Table 3. Abdominal pain was most common (66.23%) followed by indigestion (45.88%) and weight loss (43.29%), and the least common was melena (9.52%).  Table 4 showed antrum as a commonest site of involvement (51.9%) followed by body (18.6%) and fundus (16.5%), the least common being incisura (12.9%). It was also revealed that body mucosa was significantly involved in males as compared to females (*P* < 0.05). Table 5 revealed that ulcerative lesion (57.8%) was significantly common as compared to ulceroproliferative (24.9%) and polypoidal lesion (17.3%).  Table 6 revealed intestinal subtype as commonest (53.6%) and indeterminate subtype being the least common (15.1%) histological findings. Again, incidences of intestinal and indeterminate subtypes were more common in antral (61.3%, 57.1%, resp.) while diffuse subtype in fundal (37.5%) mucosa. *Helicobacter pylori* was positive in 80.09% of gastric carcinoma with the commonest incidence in intestinal subtype (89.1%) followed by diffuse type (85.2%).

## 4. Discussion

There was a spectrum of median age incidence reported in different parts of the world. In the western world, it was 71 years in the USA. In Asian countries, median ages in different countries were low. For example, in Japan it was 61 years [8], in Pakistan 48 ± 4.47 years, and in Saudi Arabia 47 years. In our study, the median age was 55 ± 11.53 years, which was near similar to the study done in South India (54.13 ± 12.53 years) and in Mizoram (male 58 and female 57 years), and male : female ratio was 2.7 : 1, where as in Mizoram, it was 2.3 : 1, [9], in Kashmir 3.3 : 1, in Saudi Arabia 2.2 : 1 [10], and in Pakistan 1.5 : 1 [11].

Our cohort showed males over age of forty years were significantly affected, which was not shown in any Indian study. In the age group of 20–39 years, Muslims were significantly affected than Hindus. It may be due to dietary indiscretion in the form of pickled food, high protein diet, and high tobacco consumption, in the form of bidi smoking and chewing [6, 7].

In astudy done by Kabir et al., abdominal pain (100%), vomiting (78%), dysphagia (24%), and weight loss (62%) were predominant symptoms pertaining gastric carcinoma [12]. In a review of 18365 patients by the American College of Surgeons, common presentations were weight loss (66.6%), abdominal pain (51.6%), nausea/vomiting (34%), anorexia (32%), and melena (20.2%). Again Qurieshi et al. showed common presenting symptoms as weight loss (35%), dyspepsia (76%), anorexia (35%), and vomiting (35.8%) [13]. Our study showed that abdominal pain (66.2%) was the commonest symptom followed by weight loss (43.3%), indigestion (45.9%), anorexia (39.9%), nausea/vomiting (34.2%), postprandial pain (29%), and melena (9.5%). Our findings were similar to the findings of the study done by Qurieshi et al. [13]. Our cohort study showed that the obstructive symptoms like postprandial abdominal pain, nausea/vomiting, and weight loss were common in fundal and obstructive variety of antral carcinoma, which was not shown in any study after thorough Medline search.

Various reports revealed progressive increase in proximal stomach cancer and concomitant decline in distal stomach cancer in the western world [14, 15]. Reports from Asian countries were conflicting. Japanese and Korean population had predominant incidence of noncardia cancer, whereas an Iranian study showed the predominance of cardia cancer. In our study antrum was mostly involved (51.9%) followed by body (18.6%) and fundus (16.4%). Gastric body was significantly involved in males (*P* < 0.05). Recent study from Kerala in India showed that though predominant site of cancer was antral mucosa, there was a trend towards proximal shift. Cherian et al. showed no change in site specificity of carcinoma of stomach in South Indian population [16]. Again Qurieshi et al. showed that in the Kashmiri population, incidences of cancer in proximal, mid, and distal stomach were 42%, 6.2%, and 45.7%, respectively [13]. Afridi et al. reported growth at cardiac end in 33%, pylorus and antrum in 40%, linitis plastica in 13.3%, and only body and body and pylorus in 6.7% of patients [11].

Macroscopically, gastric cancer has been classified into 4 types: type one: polypoidal lesion, type two: fungating lesion, type three: ulcerated lesion, and type four: infiltrating lesion on the gastric wall or linitis plastica lesion. But there is considerable overlap between the above different types. Qurieshi et al. showed 35.5% ulceroproliferative, 26% proliferative, 31% ulcerative, and 7.4% infiltrative lesions during endoscopic procedure performed in Kashmiri patients [13]. Another study done by Kabir et al. showed that ulcerative lesion was 56%, ulceroproliferative lesion 10%, and polypoidal lesion 34% [12]. In our study ulcerative lesion was 57.8% followed by ulceroproliferative lesion 24.9% and polypoidal lesion 17.3% with significant incidence of ulcerative lesion (*P* < 0.0001). 

Over the past half century, the histological classification of gastric carcinoma has been largely based on Lauren's criteria, which describes that gastric carcinoma is of two major subtypes: (1) intestinal subtype, an expansive epidemic type, corpus-dominant, more common in males, black, and elder age group most commonly associated with *Helicobacter pylori* infection and intestinal metaplasia, associated with chronic atrophic gastritis [17, 18]. Here, glandular structures are retained. This type is little invasive and has a sharp margin. It carries better prognosis. (2) Diffuse endemic cancer is more common in females and young individuals, but male to female ratio is equal [19]. It originates in the areas of pangastritis without atrophy, consisting of scattered clusters of cells with poor differentiation and deceptive margins, and much virulent [4]. Third common type, mixed type (indeterminate type), is also a common variant [20]. Relative frequencies are approximately 54% for intestinal type, 32% for diffuse type, and 15% for indeterminate type [21]. Regarding histopathological diagnosis, Afridi et al. showed that two-thirds of (66.6%) patients had diffuse subtype, 20% had intestinal subtype, and 13.3% gastric lymphoma [11]. On the other hand, Qurieshi et al. showed 38.2% poorly differentiated adenocarcinoma and 60% moderately differentiated adenocarcinoma [13]. In contrary to the Saudi study [10], our study showed that intestinal, diffuse, and indeterminate subtypes were 53.6%, 31.1%, and 15.1%, respectively, which was near identical to the study done by Kabir et al, in which intestinal, diffuse, and indeterminate type were 52%, 28%, and 20%, respectively [12]. In our study, intestinal type and indeterminate type were significantly predominant in antral mucosa (61.3% and 57.1%, resp.) whereas diffuse type was more significant in fundal mucosa (37.5%) followed by antral mucosa (33.3%).

*Helicobacter pylori*, a gm negative bacillus, colonizes in stomach mucosa and triggers the progressive sequences of gastric lesions from chronic gastritis, gastric atrophy, intestinal metaplasia, dysplasia, and finally gastric carcinogenesis [22]. Studies from different parts of the world showed linear relationship between gastric cancer and *H. pylori* infection [23, 24]. *H. pylori* infection is mainly acquired in childhood through oral ingestion, and infection may persist throughout the life [25]. Its prevalence is closely linked to socioeconomic factors such as low income and poor education and living conditions during childhood such as poor sanitation and overcrowding [26, 27]. The study in US and Japan showed increased prevalence of *Helicobacter pylori* with age; it was mainly due to birth cohort rather than late acquisition of infection [28].

Several case control studies have shown significant association between *Helicobacter pylori* and risk of gastric adenocarcinoma. The risk is 2.1 to 16.7 fold greater than that in seronegative individual [29, 30]. Effect of *H. pylori* on gastric cancer development may vary with anatomic sites. Meta-analysis of prospective cohort studies showed that *H. pylori* infection was associated with rise of noncardia gastric adenocarcinoma [31]. Most of the *H. pylori* infections are asymptomatic. Virulent strain of *H. pylori* carrying cytotoxin associated gene A (cagA) is responsible for gastric cancer in distal sites following atrophic gastritis [32, 33]. In Bangladesh, Talukdar et al. showed that the prevalence of *H. pylori* detected by CLO test and histology was 66% out of 50 cases of gastric cancer [12]. Kabir et al. showed prevalence of *H. pylori* in 71.8% of gastric cancer patients and 20% among control group, showing significant (*P* < 0.001) difference. Among the patients with intestinal type gastric cancer, *H. pylori* was present in 86.96% of cases, 50% of diffuse type and poorly differentiated type harbored *H. pylori* infection [12]. In study by Qurieshi et al., 39% of gastric cancers were *H. pylori* positive [13]. A study done by Satti et al. showed that among the patients with intestinal type gastric carcinoma, 37% were *H. pylori* positive, whereas among the patients with diffuse signet ring type gastric carcinoma, 29% were *H. pylori* positive [10]. Other Indian studies did not found any association between *H. pylori* and gastric cancer [34–37]. Seroprevalence of *H. pylori* infection in adult population in India is 55%–92%, as compared to 44% and 55% in Chinese and Japanese population, respectively. In our study, 80.09% of gastric carcinoma was *H. pylori* positive, which was contradictory to the observation in developing countries by Miwa et al. and other Indian studies [38]. 89.1% of intestinal type, 85.2% of diffuse type and 37.1% of indeterminate type of gastric cancer were *H. pylori* positive. Our observation of *H. pylori* positivity in case of intestinal type was similar to observation by Kabir et al. In comparison to other Indian studies, *H. pylori* positivity in case of gastric cancer in West Bengal was much higher.

## 5. Conclusion

In summary, in this study the male and female ratio was 2.7 : 1, religion trend Hindus : Muslim 3 : 1. Median age of Stomach cancer in Gangetic West Bengal, India, was mainly around 55 years of age. Abdominal pain indigestion and weight loss were the commonest symptoms of presentation. Anatomically antral mucosa was mostly commonly involved. Surprisingly Fundus was significantly involved in females. Histologically interstitial type was the most common subtype of adenocarcinoma and more commonly associated with *H. pylori* infection. Macroscopically ulcerative type was detected as the commonest lesion. Future target for gastric cancer prevention is to modify the risk factors, and further demographic studies and analysis for future screening will be needed.

## Figures and Tables

**Table 1:** Total number of affected patients, 462 frequency of gastric carcinoma in males and females.

Age groups (years)	Males performed (14106)	Carcinoma males (340)	Percentage of males affected	Females performed (9745)	Carcinoma females (122)	Percentage of females affected
20–39	3780	34	0.9%	2281	20	0.9%
40–59	5860	184	3.1%	4146	52	1.2%
≥60	4466	122	2.7%	3318	50	1.5%

**Table 2:** Frequency of gastric carcinoma in Hindus and Muslims.

Age groups (years)	Hindus performed (17889)	Carcinoma Hindus (310)	% of Hindus amongst affected	Muslims performed (5962)	Carcinoma Muslims (152)	% of Muslims amongst affected
20–39	5030	32	0.6%	1031	22	2.1%
40–59	7457	160	2.1%	2549	76	2.2%
≥60	5402	118	3.5%	2382	54	2.2%

**Table 3:** Percentage of occurrence of symptoms in stomach cancer (*n* = 462).

Symptom	Total incidence	Percentage (%)
Indigestion	212	45.9
Loss of appetite	184	39.9
Abdominal pain	306	66.2
Nausea/vomiting	158	34.2
Postprandial pain	134	29
Weight loss	200	43.3
Melena	44	9.5

**Table 4:** Amongst the affected cases (462)—distribution of sex and the site of carcinoma.

Sites of stomach	Male (340)	Female (122)	*P* value	95% CI	Total cases (462)	Percentage of amongst affected cases
Fundus	52 (15.3%)	24 (19.6%)	>0.05	−0.120, 0.033	76	16.4%
Body	72 (21.1%)	14 (11.5%)	<0.05	−0.017, 0.178	86	18.6%
Antrum	170 (50%)	70 (57.45%)	>0.05	−0.177, 0.030	240	51.9%
Incisura	46 (13.5%)	14 (11.5%)	>0.05	−0.049, 0.090	60	12.9%

Figures within parenthesis indicate percentages.

**Table 5:** Among the affected cases (462), distribution of type of lesions.

Types of lesion	Number of cases	% affected	Chi-square test	*P* value
Ulcerative lesion	267	57.8	90.765	<0.0001
Ulceroproliferative lesion	115	24.9		
Polypoidal lesion	80	17.3		

**Table 6:** Comparison of incidence of histopathological subtypes and *H. pylori* with sites of gastric carcinoma.

Cell types	Fundus (76)	Body (86)	Antrum (240)	Incisura (60)	% of involvement of each subtype in total cases (462)	*H. pylori* positivity according to subtype
Intestinal type (248)	18 (7.2%)	42 (16.9%)	152 (61.3%)	36 (14.6%)	53.6%	221 (89.1%)
Diffuse type (144)	54 (37.5%)	28 (19.4%)	48 (33.3%)	14 (9.8%)	31.1%	123 (85.2%)
Indeterminate type (70)	4 (5.7%)	16 (22.9%)	40 (57.1%)	10 (14.3%)	15.1%	26 (37.1%)

Figures within the parenthesis indicate percentages.
